# Patient-derived *AMOTL1* mutations lead to defective cell migration and tissue development

**DOI:** 10.1042/BSR20250149

**Published:** 2026-05-11

**Authors:** Jiaqian Luo, Ruxin Jin, Fang Geng, Yunying Wang, Yuwen Zhu, Wenqiang Gao, Wei Gao, Jian Li, Yaming Jiu, Ruilin Zhang, Fa-Xing Yu, Yu Wang

**Affiliations:** 1 Institute of Pediatrics, Children’s Hospital of Fudan University, and Shanghai Key Laboratory of Medical Epigenetics, International Co-laboratory of Medical Epigenetics and Metabolism, Institutes of Biomedical Sciences, Shanghai Medical College, Fudan University, Shanghai, China; 2 School of Basic Medical Sciences, Wuhan University, Wuhan, China; 3 School of Life Sciences, Fudan University, Shanghai 200433, China; 4 Shanghai Institute of Materia Medica, Chinese Academy of Sciences, Shanghai, China; 5 University of Chinese Academy of Sciences, Beijing, China

**Keywords:** Angiomotin-like 1, proteasomes, RNF146, TNKS1/2

## Abstract

Angiomotin-like 1 (AMOTL1), by regulating cell–cell junctions, cell polarity, and cell migration, plays a critical role in organogenesis and development. Recently, multiple studies have identified two hotspot mutations in *AMOTL1*, Arg157 (R157) and Pro160 (P160), in more than ten distinct families presenting with a spectrum of congenital defects, including facial dysmorphisms and cardiac abnormalities. However, the underlying pathogenic mechanism remains elusive. R157 and P160 are located in the highly conserved Tankyrase-binding motif (TBM) of AMOTL1. Here, we show that both the R157C and P160L mutants fail to interact with Tankyrase 1/2 and Ring finger protein 146, rendering them unable to undergo poly ADP-ribosylation, ubiquitination, and subsequent proteasomal degradation. As a result, these mutants are significantly stabilized and accumulate in the cytoplasm. Accumulated AMOTL1 mutants, in turn, disrupt cell junctions and focal adhesions, thereby inhibiting both the velocity and persistence of cell migration. Furthermore, during zebrafish embryonic development, expression of the R157C mutant leads to craniofacial malformations and defects in cardiac function and skeletal muscle. Our study confirms the role of *AMOTL1* mutations in tissue development and uncovers the pathogenic mechanism at both molecular and cellular levels.

## Introduction

Angiomotin (AMOT), Angiomotin-like 1 (AMOTL1), and Angiomotin-like 2 (AMOTL2) are Motin family proteins (Motins) involved in angiogenesis, organogenesis, and tumorigenesis [1,2]. Motins are recognized as adaptor proteins regulating cell polarity and cell migration [3–6]. Due to sequence and structural homology, different Motins appear to have functional redundancy despite their distinct expression patterns in human tissues [1]. Recently, Motins have been identified as components of the Hippo signaling pathway, which plays a pivotal role in cell proliferation and tumorigenesis [7–11].

Motins colocalize with cell–cell junctions and the actin cytoskeleton in endothelial and epithelial cells [8,12,13]. Phosphorylation of Motins by large tumor suppressor 1/2 (LATS1/2), the central kinases in the Hippo pathway, affects the subcellular localization of Motins, leading to cytoskeletal rearrangement and disruption of cell–cell junctions, ultimately impairing cell migration [8,10,14]. In addition, the protein stability of Motins is regulated by the ubiquitin–proteasome system (UPS), and multiple E3 ligases, including Ring finger protein 146 (RNF146), label Motins for degradation [15–18]. The activity of RNF146 depends on Tankyrase 1/2 (TNKS1/2) (also known as PARP5A/B), as Motins are first modified by TNKS1/2 through poly ADP-ribosylation (PARylation) and subsequently ubiquitinated by RNF146 [17,19–21]. We have recently identified a signaling pathway in which AMOT undergoes precise, regulated cleavage, a process essential for optimal collective cell migration (CCM) and angiogenesis in both zebrafish and mice. Mechanistically, upon LPA stimulation, NF2 orchestrates the recruitment of TNKS1/2, RNF146, and AMOT, leading to AMOT’s PARylation and ubiquitination. Ubiquitinated AMOT may either be targeted for degradation by the UPS or recognized by DDI2, which specifically cleaves it between amino acids F131 and Y132. Following cleavage, the C-terminal fragment of AMOT (AMOT-CT) translocates to the cell–matrix interface, where it promotes focal adhesion (FA) maturation, generates traction force, and induces leader cell formation [5,22,23].

AMOTL1 was discovered in a screen for tight junction-associated proteins and initially named junction-enriched and -associated protein, later renamed Angiomotin-like 1 due to its similarity to AMOT [24]. Several studies report that AMOTL1 mediates embryonic angiogenesis in zebrafish and postnatal retinal vascularization in mice [4,25,26]. The human *AMOTL1* gene is located on chromosome 11q21. Recent genomic analyses have identified critical mutations in the *AMOTL1* gene, with significant implications for developmental abnormalities. A *de novo* missense variant (chr11:94532825; c.469C>T; p.Arg157Cys, R157C) was identified in a family with congenital defects [27], while another pathogenic mutation (c.479C>T; p.Pro160Leu, P160L) was found in a family exhibiting vertebral, anal, tracheoesophageal, radial, and renal (VACTERL)-like birth abnormalities [28]. Further *in silico* predictions with multiple bioinformatic models supported the pathogenicity of both R157C and P160L variants [27,28]. Subsequent cohort studies identified several heterozygous *AMOTL1* variants, primarily affecting amino acids 157–161, and linked them to phenotypes such as orofacial clefting, congenital heart disease, tall stature, auricular anomalies, and gastrointestinal manifestations [29]. These studies indicate a pivotal role for AMOTL1 in development. However, the pathogenic mechanisms underlying *AMOTL1* mutations remain elusive.

In the current study, we demonstrate that AMOTL1 proteins encoded by patient-derived mutants are stabilized due to defective interactions with TNKS1/2 and RNF146, and the pathological accumulation of AMOTL1 mutants in the cytoplasm impairs cell polarity and migration. In zebrafish models, we demonstrate the important role of *AMOTL1* mutation in embryonic development of various organs, including orofacial structure growth and the development of the heart and skeletal muscle.

## Results

### R157C and P160L AMOTL1 mutants are stabilized

**Figure 1:**
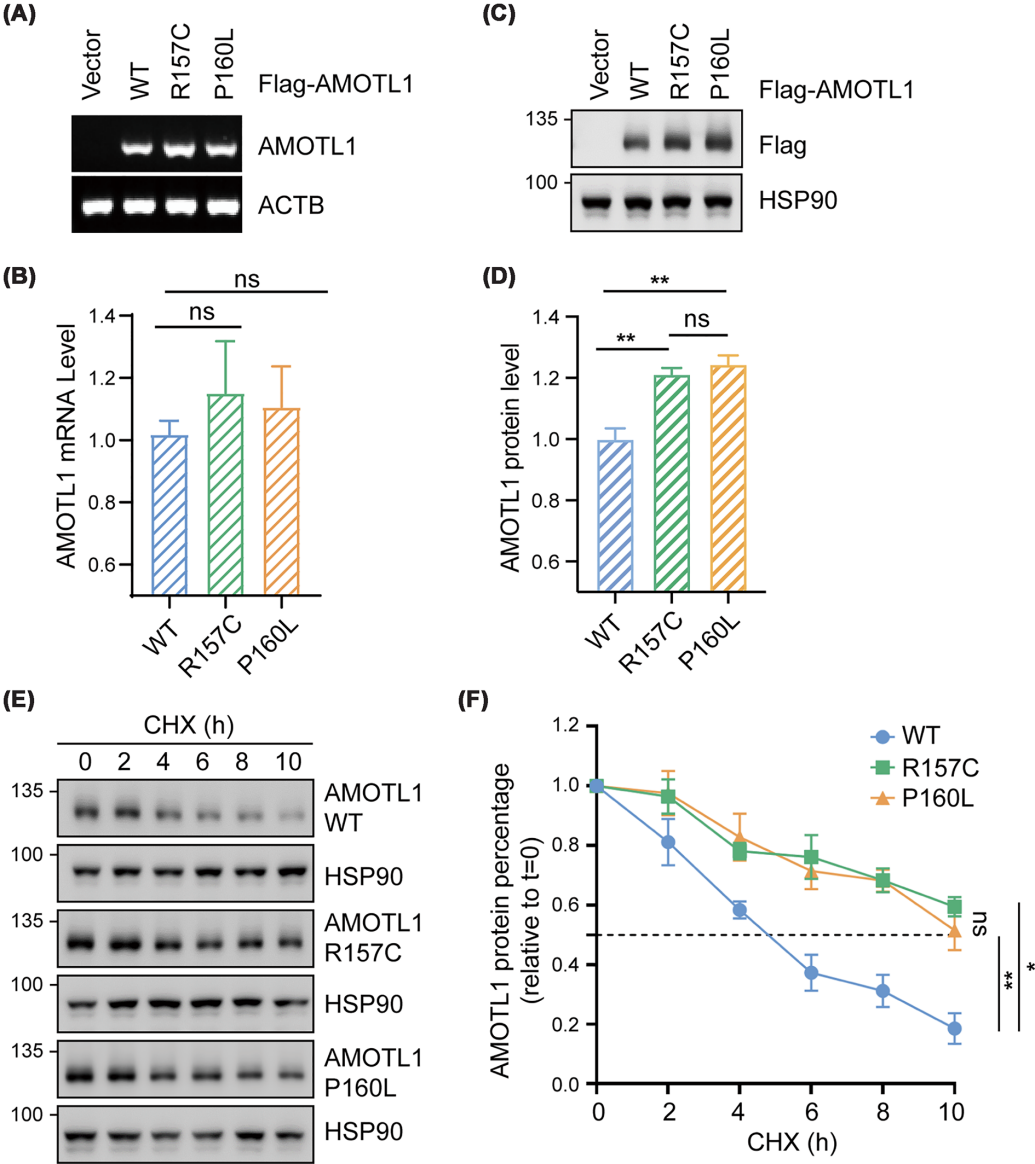
R157C and P160L mutations lead to AMOTL1 protein stabilization and accumulation (**A**,** B**)*AMOTL1* mRNA levels in HEK293A cells overexpressing Vector, WT, R157C, or P160L AMOTL1 were analyzed by RT-PCR (**A**) and quantitative real-time PCR (**B**), *n* = 3. The mRNA levels of *ACTB* and glyceraldehyde-3-phosphate dehydrogenase (*GAPDH*) were used as internal controls in panels (**A, B**), respectively. (**C**,** D**) AMOTL1 protein levels in HEK293A cells overexpressing Vector, WT, R157C, or P160L AMOTL1. Representative immunoblotting results (**C**) and quantification (**D**) were shown, *n* = 3. Heat shock protein 90 (HSP90) expression was used as the loading control. (**E**,** F**) Protein stability of WT, R157C, or P160L AMOTL1. HEK293T cells transfected with Flag-tagged WT, R157C, or P160L AMOTL1 were treated with 100 μg/ml cycloheximide (CHX) to inhibit new protein synthesis over a 10 h time course. Cell lysates were collected at indicated time points and subjected to immunoblotting, with HSP90 used as the internal loading control (**E**). The protein levels of AMOTL1 were quantified (**F**), *n* = 4. The dotted line indicates 50% of the initial normalized AMOTL1 protein signal. Data are presented as mean ± SEM. *P <0.05, **P <0.01, ***P <0.001, and ****P <0.0001; ns: no significant difference (two-tailed Student’s *t*-test).

To investigate the function of AMOTL1 mutants, we established HEK293A cell lines stably expressing Flag-tagged wild-type (WT), R157C, or P160L AMOTL1. Afterward, mRNA and protein levels of AMOTL1 were assessed by RT-PCR and immunoblotting, respectively. While no significant difference in *AMOTL1* mRNA levels was detected (Figure 1A,B), the protein levels of both AMOTL1 mutants were significantly higher than those of the WT protein (Figure 1C,D). These data indicate that R157C and P160L mutations lead to increased AMOTL1 abundance, which most likely occurs via a post-transcriptional mechanism.

To determine whether protein turnover of WT and mutant AMOTL1 differs, we treated cells expressing different AMOTL1 with CHX to block protein translation and monitored AMOTL1 protein levels over time. As shown in Figure 1E,F, WT AMOTL1 was gradually degraded, whereas the stability of the R157C and P160L mutants was higher, with half-lives at least twice as long as that of WT AMOTL1. Hence, the protein stability of AMOTL1 is significantly increased when R157C and P160L mutations are introduced.

### R157C AMOTL1 abrogates binding to TNKS1/2 and RNF146, blocking PARylation and ubiquitination

**Figure 2:**
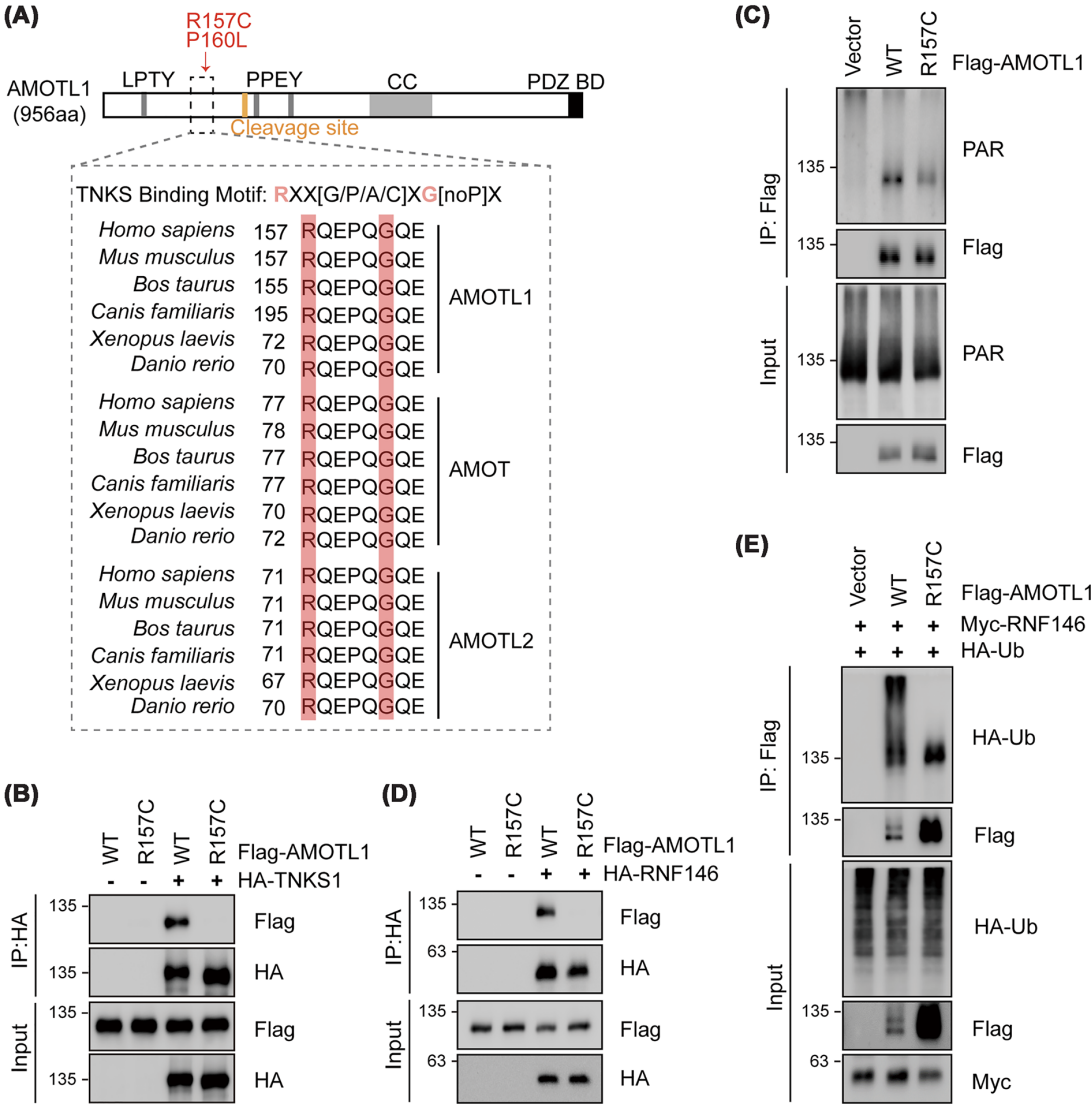
R157C AMOTL1 is not modified by PARylation and ubiquitination (**A**) R157 and P160 are key residues in the evolutionarily conserved TBM of Motins. Schematic representation of human AMOTL1 domains and alignment of TBM sequences from different Motins. Key amino acids of TBM are highlighted. PPxY motifs and putative DDI2 cleavage site are indicated. CC: coiled‐coil domain and PDZ BD: PDZ binding motif. (**B**) R157C AMOTL1 is unable to interact with TNKS1. HA-tagged TNKS1 was co-expressed with Flag-tagged AMOTL1 (WT or R157C) in HEK293A cells; proteins or protein complexes were co-immunoprecipitated with anti-Flag beads and analyzed by immunoblotting. (**C**) PARylation of R157C AMOTL1 is markedly reduced. HEK293T cells were transfected with Flag-tagged AMOTL1 (WT or R157C). Immunoprecipitated AMOTL1 proteins were subjected to immunoblotting using antibodies recognizing poly ADP-ribose (PAR). (**D**) R157C AMOTL1 is unable to interact with RNF146. (**E**) Ubiquitination of R157C AMOTL1 is inhibited. HEK293T cells were transfected with Flag-tagged AMOTL1 (WT or R157C), Myc-tagged RNF146, and HA-tagged ubiquitin (Ub). Cells were treated with 1 μM proteasome inhibitor bortezomib (BTZ) for 6 h to inhibit proteasomal degradation. Immunoprecipitated AMOTL1 proteins were then subjected to anti-HA immunoblotting to assess ubiquitination.

When examining the amino acid sequence of AMOTL1, we noticed that both R157 and P160 are key residues within the highly conserved Tankyrase-binding motif (TBM), defined as RXX[G/P/A/C]XG[noP]X, where ‘X’ represents any amino acid (Figure 2A) [30,31]. The interaction between Motins and TNKS1/2 via the TBM is critical for the protein degradation of Motins [19,31]. Therefore, the R157C and P160L mutations are likely to impair the interaction with TNKS1/2, thereby preventing the initiation of TNKS-dependent protein degradation.

To assess whether R157C and P160L AMOTL1 can interact with TNKS1/2, we ectopically co-expressed HA-tagged TNKS1 and Flag-tagged AMOTL1 in HEK293A cells and performed co-immunoprecipitation. Because the R157C mutant was more stable than WT AMOTL1, we titrated the respective plasmids to ensure comparable expression levels of WT and mutant AMOTL1 (Figure 2B). WT AMOTL1 was effectively co-precipitated with TNKS1, whereas the R157C and P160L mutants failed to interact with TNKS1 (Figure 2B and Supplementary Figure S1A). Given that TNKS1/2 mediate PARylation of their substrates, including AMOTL1 [19,21], we next examined whether defective TNKS1/2 binding affects PARylation status. Following expression and immunoprecipitation of WT, R157C, and P160L AMOTL1, immunoblotting with an anti-PAR/pADPr antibody showed a marked reduction in PARylation of the R157C and P160L mutants (Figure 2C and Supplementary Figure S1B).

TNKS1/2-dependent PARylation is required for the recruitment of the E3 ligase RNF146, which subsequently ubiquitinates substrates to direct them for proteasomal degradation [32]. Consistent with impaired PARylation, R157C and P160L AMOTL1 displayed greatly reduced interaction with RNF146 (Figure 2D and Supplementary Figure S1C). To further assess the impact on ubiquitination, we co-expressed WT or R157C AMOTL1 together with HA-tagged ubiquitin and analyzed ubiquitin conjugation following AMOTL1 immunoprecipitation. Despite the increased abundance of the R157C mutant, its ubiquitination was substantially decreased (Figure 2E and Supplementary Figure S1B).

Consistently, pharmacological inhibition of TNKS1/2 using XAV-939 led to marked accumulation of AMOTL1, accompanied by reduced PARylation and ubiquitination (Supplementary Figure S1B,D,E), thereby recapitulating the stabilization observed in cells expressing the R157C and P160L mutants. Together, these data demonstrate that mutations within the TBM of AMOTL1, such as R157C and P160L, impede its interactions with TNKS1/2 and RNF146, resulting in diminished PARylation and ubiquitination and consequently reduced proteasomal degradation.

### R157C AMOTL1 disrupts cell–cell and cell–matrix adhesions

**Figure 3:**
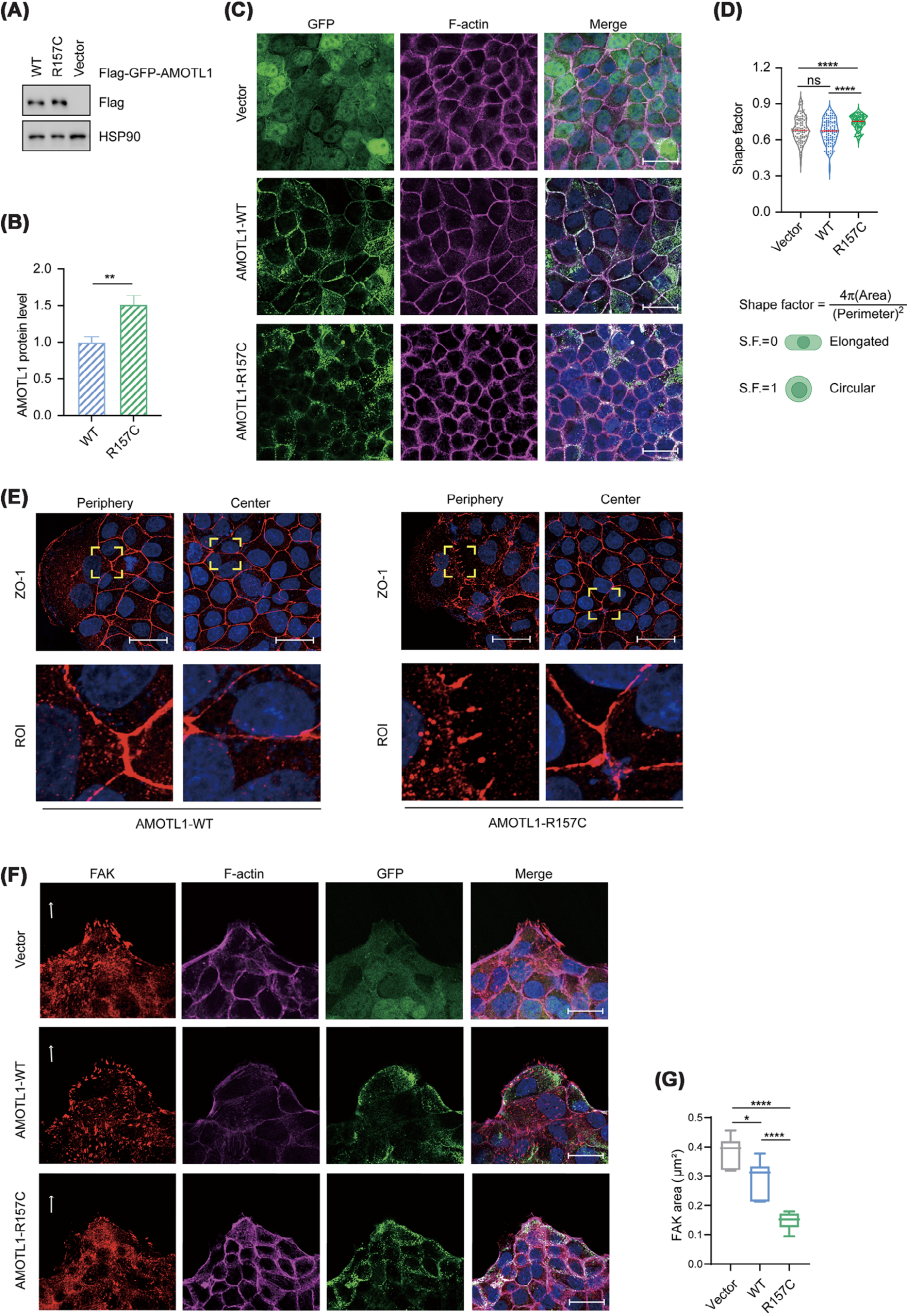
R157C AMOTL1 expression disrupts cell–cell and cell–ECM adhesions (**A**,** B**) Expression levels of WT and R157C AMOTL1 in stable cell lines. Immunoblotting of Flag and HSP90 (A) and quantification (B) are shown, *n* = 4. (**C**) Subcellular localization of WT and R157C AMOTL1. Monolayer MDCK cells expressing GFP, WT AMOTL1, or R157C AMOTL1 were stained for filamentous actin (F-actin) (magenta) and DAPI (blue). Scale bar = 10 μm. (**D**) Quantification of cell shape using the shape factor. Higher shape factor values indicate a more circular morphology, whereas lower values reflect a more polygonal shape. Each dot represents an individual cell. (**E**) Disruption of tight junctions in MDCK cells by R157C AMOTL1 expression. MDCK cells expressing WT or R157C AMOTL1 were cultured to form a monolayer, fixed, and stained for ZO-1 antibody (red) and DAPI (blue). Cells at the periphery and center of the monolayer are shown, with regions of interest (ROI) presented at higher resolution. Scale bar = 10 μm. (**F**) Disruption of FAs by R157C AMOTL1 expression. MDCK cells expressing GFP control (Vector), WT AMOTL1, or R157C AMOTL1 were cultured into monolayers. Cells were fixed and stained for focal adhesion kinase (FAK) (red), F-actin (magenta), and DAPI (blue). Direction of cell migration is indicated by white arrows. Scale bar = 10 μm. (**G**) Quantification of FAK size at the leading front of MDCK cells overexpressing WT or R157C AMOTL1. Data represent FAK staining from independent confocal fields; a total of *n* = 99 (Vector), 160 (WT AMOTL1), and 77 (R157C AMOTL1) FAs were quantified. The box-and-whisker plot represents the distribution of data across the indicated groups. Data are presented as the mean ± SEM. *P <0.05, **P <0.01, ***P <0.001, and ****P <0.0001; ns: no significant difference (two-tailed Student’s *t*-test).

Motins mainly localize to cell–cell junctions and the actin cytoskeleton and are involved in the maintenance of cell polarity and cell–cell adhesion [25]. To investigate the impact of *AMOTL1* mutations on these processes, we expressed green fluorescent protein (GFP)-tagged WT and R157C AMOTL1 in Madin–Darby canine kidney (MDCK) epithelial cells by lentiviral infection and compared their subcellular localization and effects on the integrity of cell–cell junction. Even though fewer R157C AMOTL1 viruses were used to infect cells, expression of the AMOTL1 mutant was slightly higher than that of WT AMOTL1, consistent with reduced proteasomal degradation resulting from impaired PARylation and ubiquitination (Figure 3A,B).

We then analyzed the subcellular localization of WT and R157C AMOTL1 in MDCK cells. Both WT and R157C AMOTL1 exhibited a dominant subcellular localization to cell junctions, whereas the R157C mutant showed more punctate staining in the cytoplasm (Figure 3C). Consistent with this observation, in an independent cell type, both R157C and P160L AMOTL1 mutants also exhibited punctate cytoplasmic accumulation in NIH-3T3 cells (Supplementary Figure S2A). The deficiency in PARylation and ubiquitin-mediated degradation of both mutants likely disrupts their junctional localization, leading to notable cytoplasmic accumulation and puncta formation. Notably, both R157C and P160L AMOTL1 mutants failed to co-localize with actin stress fibers, in contrast with WT AMOTL1. This mislocalization likely compromises AMOTL1-mediated regulation of both cell–cell and cell–matrix adhesions (Supplementary Figure S2A).

Typically, epithelial cells in a dense monolayer are polarized and maintain a polygonal shape; however, in the presence of R157C AMOTL1, cells displayed a more circular morphology (Figure 3C,D), possibly reflecting impaired adhesions and dysregulated mechanical forces between cells and their environment. To determine whether these morphological changes were associated with altered junctional organization, we subsequently examined the tight junction in migrating MDCK cells. In the center of the monolayer, cells overexpressing both WT and R157C AMOTL1 exhibited intact junctions, as shown by staining with the tight junction marker zonula occludens-1 (ZO-1). However, at the leading edge of the migrating cell monolayer, cells expressing R157C AMOTL1 exhibited more punctate ZO-1 staining within the cytoplasm, along with less organized and significantly disrupted cell junctions (Figure 3E). AMOT proteins have been shown to associate with the tight junction and Crumbs complex proteins in epithelial cells [33]. A previous study showed that ablation of RNF146/TNKS1/2, or overexpression of AMOT, led to the relocation of PALS1 (a Crumbs complex component) from the junction to internal puncta, which disturbs the function of tight junctions [17]. Hence, the cytoplasmic accumulation of AMOTL1 with the R157C mutation likely disrupts tight junction integrity by altering the localization of junctional proteins.

In addition to cell–cell adhesions, epithelial cells also interact with extracellular matrix (ECM) components to form cell–matrix adhesions. For instance, integrins on the plasma membrane interact with extracellular fibronectin and facilitate the formation of FA, which play an important role in cell migration. We recently reported that NF2 facilitates the recruitment of TNKS1/2, RNF146, and AMOT, resulting in AMOT’s PARylation and ubiquitination. The ubiquitinated AMOT is subsequently recognized and specifically cleaved by DDI2. The resulting cleavage product, AMOT-CT, translocates from cell junctions to stress fibers and FAs, where it promotes FA maturation and CCM. This mechanism is likely conserved in AMOTL1 [5,22,23]; however, the R157C mutation disrupts TNKS1/2 binding, resulting in deficient PARylation and ubiquitination, which in turn prevent DDI2 recognition and cleavage of AMOTL1 (Supplementary Figure S2B,C), thereby impairing its ability to promote FA maturation. To assess the functional impact of the AMOTL1 R157C mutant, we stained FAK, a key FA component, to determine the size and morphology of FAs (Figure 3F). In control cells and cells expressing WT AMOTL1, we observed large FA clusters that were enriched at the migration front. In contrast, FAK signals were diffuse in cells expressing R157C AMOTL1, and the number and size of large FA clusters were significantly decreased, indicating a defect in FA maturation (Figure 3F,G). These data suggest that the R157C AMOTL1 mutant also disrupts the structure of cell–ECM adhesions.

In summary, the inability of the R157C and P160L mutants to bind TNKS1/2 leads to deficient PARylation and ubiquitination, likely resulting in a loss of membrane localization and abnormal accumulation in the cytoplasm, thereby disrupting cell–cell junctions. Moreover, the failure of the R157C mutant to undergo DDI2-dependent cleavage impairs its functionality in promoting FA maturation and compromises cell–matrix adhesions.

### R157C AMOTL1 inhibits cell migration

**Figure 4:**
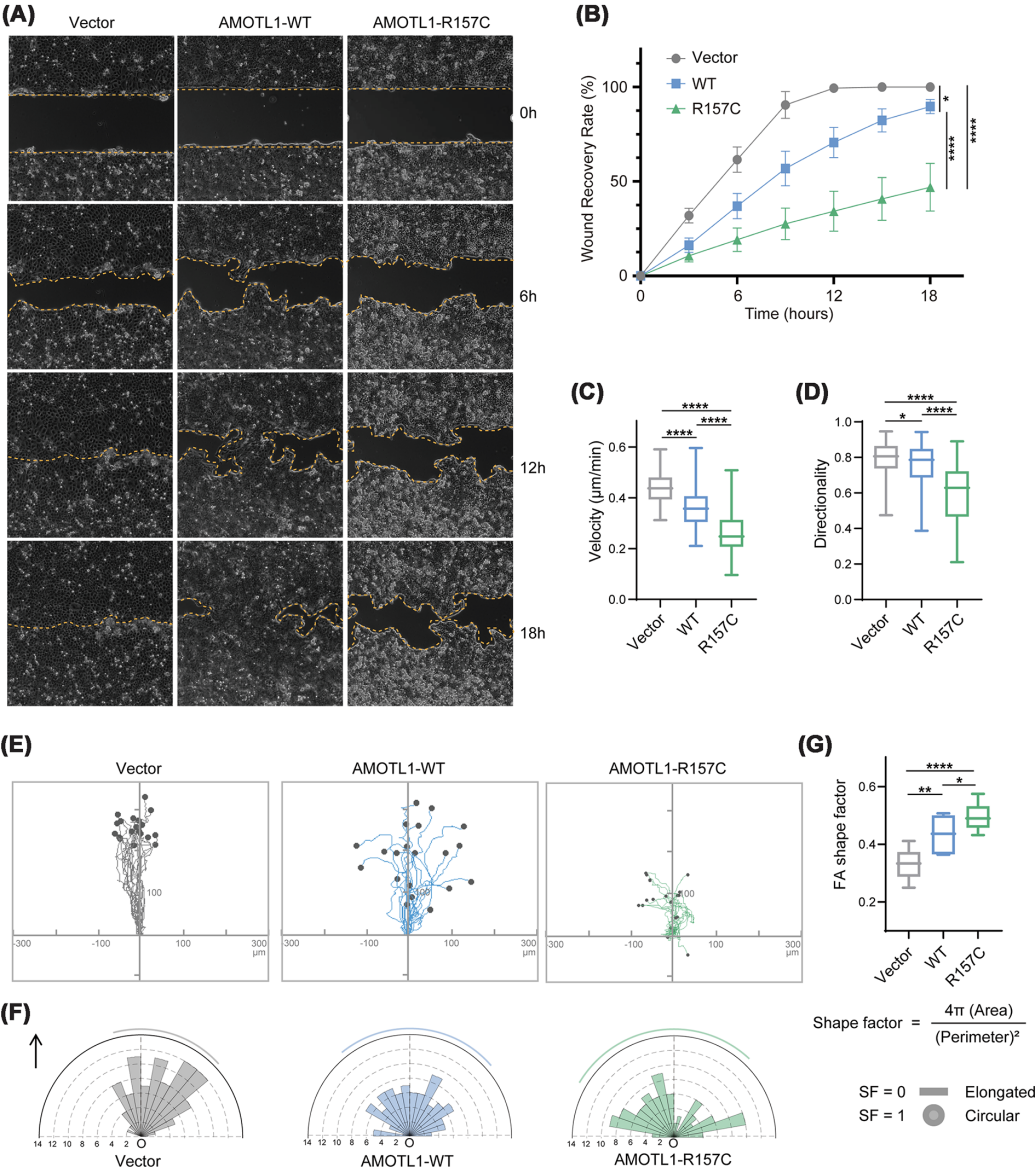
R157C AMOTL1 expression inhibits cell migration (**A**,** B**) AMOTL1 expression represses wound healing. Control MDCK cells and cells overexpressing WT or R157C AMOTL1 were cultured into confluent monolayers, and an open space (wound) was created by removing a physical block to initiate cell migration. Cell migration was monitored for 18 h by live-cell imaging. Images were captured at indicated time points (A). Wound recovery rates were quantified (B) by measuring the wound area at each time point and presented as a percentage of the initial (0 h) open area, *n* = 5. Data are presented as mean ± SEM. (**C**,** D**) Analysis of cell migration pattern in MDCK cells overexpressing Vector, WT, or R157C AMOTL1. Cell migration velocity (C) and directional persistence (directionality, D) were calculated with *n* = 110 (Vector), 142 (WT AMOTL1), and 142 (R157C AMOTL1) cells from three independent experiments. Box-and-whisker plot represents the distribution of data across the indicated groups. (**E**) Representative migration trajectories of randomly selected cells from each group, *n* = 30. (**F**) Rose diagrams showing the distribution of the orientation angles of FAs. FAs were identified by FAK staining. The magnitude of each bar shows the number of FAs with the indicated orientation. O: origin. The arrow indicates direction perpendicular to the edge of open space shown in panel (A). The outermost curved line in each diagram indicates SD. *n* = 99 (Vector), 160 (WT AMOTL1), and 77 (R157C AMOTL1) FAs were analyzed from three independent experiments. (**G**) Shape factors of FAs. FA size and perimeter were determined, and the shape factor was defined by the formula shown. Data were analyzed from *n* = 99 (Vector), 160 (WT AMOTL1), and 77 (R157C AMOTL1) FAs. Box-and-whisker plot represents the distribution of data across the indicated groups. *P <0.05, **P <0.01, ***P <0.001, and ****P <0.0001; ns: no significant difference (two-tailed Student’s *t*-test).

Motins are involved in regulating cell migration [5,34]. During cell migration, FAs serve as major determinants of directionality and speed [35]. Since FAs are severely disrupted upon R157C AMOTL1 expression, we then investigated how this mutation affects cell migration. In a wound healing assay, in which a monolayer of MDCK cells collectively migrates toward an open space, the speed and direction of cell movement can be continuously monitored by live-cell microscopy. We observed that the expression of both WT and R157C AMOTL1 inhibited cell migration, as indicated by slower wound closure rates (Figure 4A,B; Supplementary Figure S2D; Supplementary Video S1). We also performed single-cell tracking for cells in the front 3–4 rows, and the results indicated that overexpression of AMOTL1, and particularly the R157C mutant, reduced both the velocity and persistence (an indicator of migration directionality) of cell migration (Figure 4C,D). Based on single-cell trajectory analyses, we found that control cells predominantly migrated in a direction perpendicular to the wound leading edge, whereas cells expressing AMOTL1 exhibited pronounced displacement parallel to the leading edge (Figure 4E). We next examined the morphology and orientation of FAs during migration. In contrast with the elongated FAs observed in control cells, FAs in AMOTL1-expressing cells appeared more rounded, and their orientations were highly correlated with the migration trajectories of individual cells (Figure 4F,G). Notably, the effect of R157C AMOTL1 on cell migration was substantially more pronounced than that of WT AMOTL1 (Figure 4). Collectively, these findings demonstrate a critical role for *AMOTL1* mutation in regulating directional cell migration.

### R157C AMOTL1 impairs embryonic development in zebrafish

**Figure 5:**
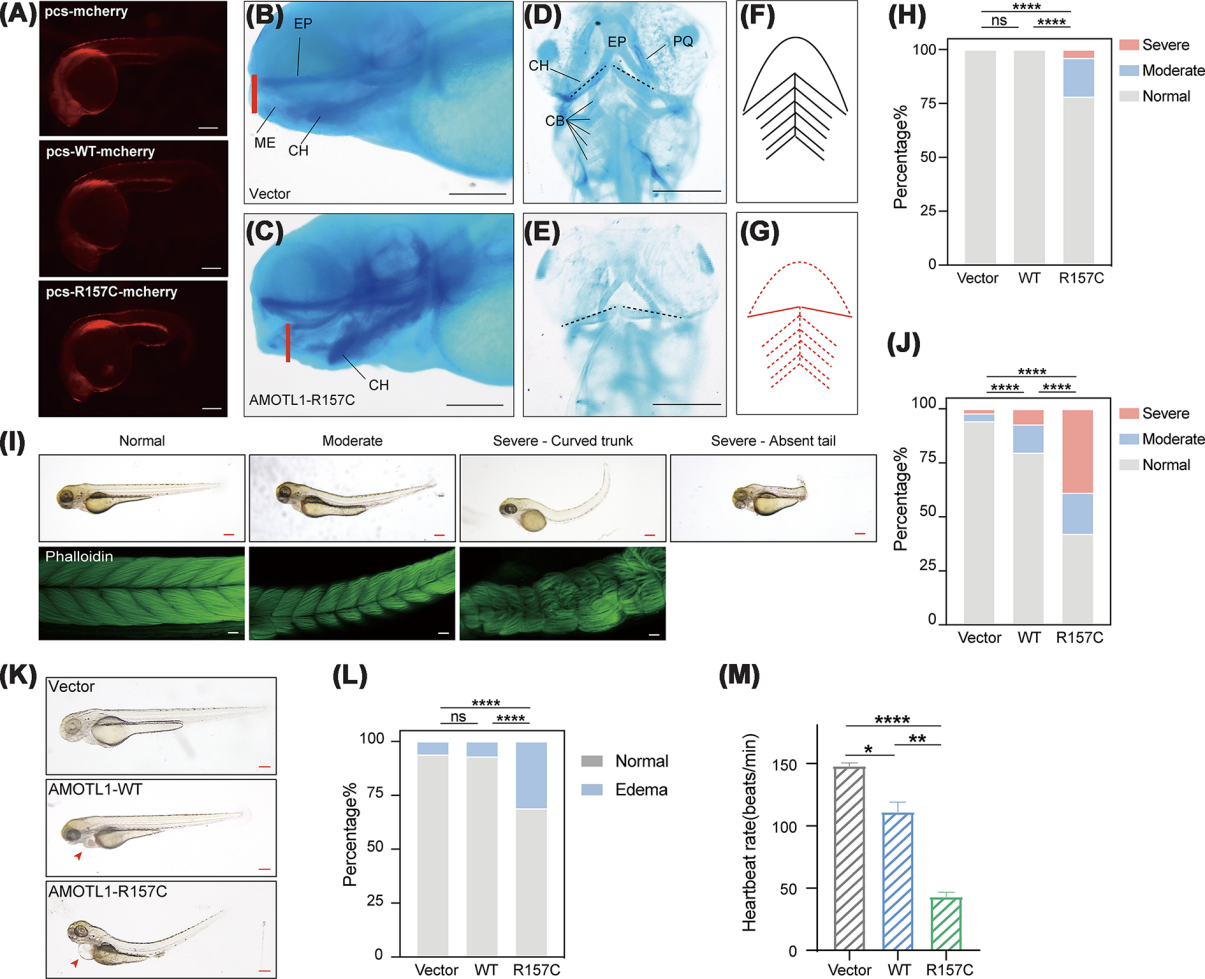
Expression of WT or R157C AMOTL1 leads to developmental defects in zebrafish (**A**) Fluorescent signal detected in zebrafish embryos injected with mCherry control or mCherry-tagged WT or R157C *AMOTL1* mRNA at 1 day post-fertilization (dpf). (**B**–**G**) Alcian blue staining of zebrafish expressing mCherry or mCherry–AMOTL1-R157C at 3 dpf. Lateral views of zebrafish expressing AMOTL1-R157C indicated defective craniofacial cartilage structures. Red lines represent lower jaw retrusion, and positions of Meckel’s cartilage (ME) and ethmoid plate (EP) are indicated (B, C). Dashed lines indicate the malformed ceratohyal (CH) in AMOTL1-expressing zebrafish (D, E). Schematic presentations of analyzed zebrafish craniofacial cartilage are shown, with solid lines indicating present structures and dashed lines indicating absent structures in AMOTL1-expressing zebrafish, including ceratobranchial (CB) and palatoquadrate (PQ) (F, G). (**H**) Quantification of craniofacial cartilage phenotypes in embryos injected with Vector, WT *AMOTL1*, or *AMOTL1* R157C. Embryos were classified as normal, moderate, or severe based on craniofacial cartilage morphology. Statistical significance was determined using the Chi-square test for trend. Representative images are also presented in Supplementary Figure S3 to illustrate the criteria for phenotype classification. Vector (*n* = 38), WT AMOTL1 (*n* = 43), and R157C AMOTL1 (*n* = 50). (**I**,** J**) Representative images of trunk curvature phenotype in zebrafish injected with control or *AMOTL1* at 3 dpf. Trunk morphology was classified into three grades: normal, moderate (mild trunk curvature), and severe (pronounced curvature or absence of tail). Embryos are shown in lateral view. Phalloidin staining (muscle) is also shown. The percentage of embryos exhibiting each morphological category for Vector (*n* = 246), WT AMOTL1 (*n* = 237), and R157C AMOTL1 (*n* = 232) is presented in panel (J). Phenotype distribution was compared across groups using a Chi-square test for trend. (**K**) Representative images showing cardiac phenotypes in zebrafish embryos injected with control, WT *AMOTL1*, or *AMOTL1* R157C RNA at 3 dpf. Arrowheads indicate pericardial edema. (**L**) Quantification of cardiac edema phenotypes in zebrafish embryos. Vector (*n* = 48), WT AMOTL1 (*n* = 56), and R157C AMOTL1 (*n* = 77). (**M**) Heartbeat rate measurements in zebrafish embryos at 3.5 dpf. Heart rate was quantified from embryos with normal body morphology to avoid potential interference from severe developmental abnormalities, *n* = 5. Data are presented as mean ± SEM. *P <0.05, **P <0.01, ***P <0.001, and ****P <0.0001 (two-tailed Student’s *t*-test). Scale bar for phalloidin staining in panel (I) is 10 μm, and others are 0.2 mm.

Our data so far suggest that the accumulation of mutant AMOTL1 disrupts cell–cell junctions and FAs, thus inhibiting cell migration. In vertebrates, orofacial architecture is formed by cranial neural crest cells (CNCCs), a highly migratory cell population that forms facial prominences and ultimately gives rise to craniofacial cartilage [36]. VACTERL-like malformations observed in several affected patients, including vertebral, cardiac, and anorectal abnormalities, are primarily midline derivatives [29,37]; hence, we hypothesized that the inhibition of cell migration by AMOTL1 mutants could disturb orofacial morphogenesis. Zebrafish is an ideal model organism for studying embryonic development in vertebrates [36,38]. To assess the developmental impact of *AMOTL1* mutations, we injected mRNA encoding mCherry-tagged WT or R157C *AMOTL1* into zebrafish embryos and examined their phenotypes for congenital defects (Figure 5A).

Defects in zebrafish craniofacial structure were observed following Alcian blue cartilage staining at 3 dpf (days post fertilization). Compared with control embryos, ectopic expression of AMOTL1 R157C resulted in abnormalities in craniofacial cartilage organization, including a shortened and retrusive lower jaw (Figure 5B,C). Ventral views further revealed malformed CH cartilages and underdeveloped anterior and branchial arches. In particular, the CH was linear in shape, and staining of the ME, PQ, and CB cartilages was reduced or absent in embryos with more severe phenotypes (Figure 5D–H and Supplementary Figure S3). These data suggest that up-regulated expression of AMOTL1 impairs craniofacial development in zebrafish and results in mandibular hypoplasia, a feature that is consistent with craniofacial abnormalities reported in patients.

Zebrafish expressing ectopic AMOTL1 exhibited additional developmental abnormalities that overlap with features reported in patients carrying *AMOTL1* mutations. For example, curved trunks and loss of the tail were observed in zebrafish, consistent with skeletal abnormalities reported in patients, including scoliosis and other skeletal deformities (Figure 5I,J). These defects might be associated with disrupted F-actin organization in skeletal muscle, as revealed by phalloidin staining (Figure 5I). Moreover, ectopic expression of AMOTL1 resulted in defective cardiac development in zebrafish, manifested as pericardial edema and decreased heartbeat rates (Figure 5K–M). It is noteworthy that, when compared with WT AMOTL1 expression, both the penetrance and severity of defects in trunk and heart development were more pronounced in zebrafish expressing R157C AMOTL1 (Figure 5I–M). Taken together, this *in vivo* evidence indicates a direct pathogenic role of *AMOTL1* mutations in the development of various organs.

## Discussion

To ensure normal organogenesis, different cells are required to reach the right location at the right time; hence, cell migration plays a critical role in embryonic development. Motins are known to regulate cell migration by interacting with cell–cell junctions and the actin cytoskeleton [5,12,39,40]. Recent studies have discovered R157C and P160L mutations in human *AMOTL1* through whole-exome sequencing of more than ten patient families presenting with a spectrum of developmental defects [27–29], suggesting that *AMOTL1* mutations are involved in congenital disorders. Using zebrafish as a model, we show that expression of AMOTL1 mutants induces craniofacial malformations, pericardial edema, bradycardia, and trunk curvature, indicative of developmental defects and partially overlapping with features reported in patients. Mechanistically, we have revealed that *AMOTL1* mutations lead to accumulation of AMOTL1 protein, disruption of cell–cell junctions and the cytoskeleton, and inhibition of cell migration. The impaired cell migration likely contributes to developmental defects associated with *AMOTL1* mutations.

**Figure 6:**
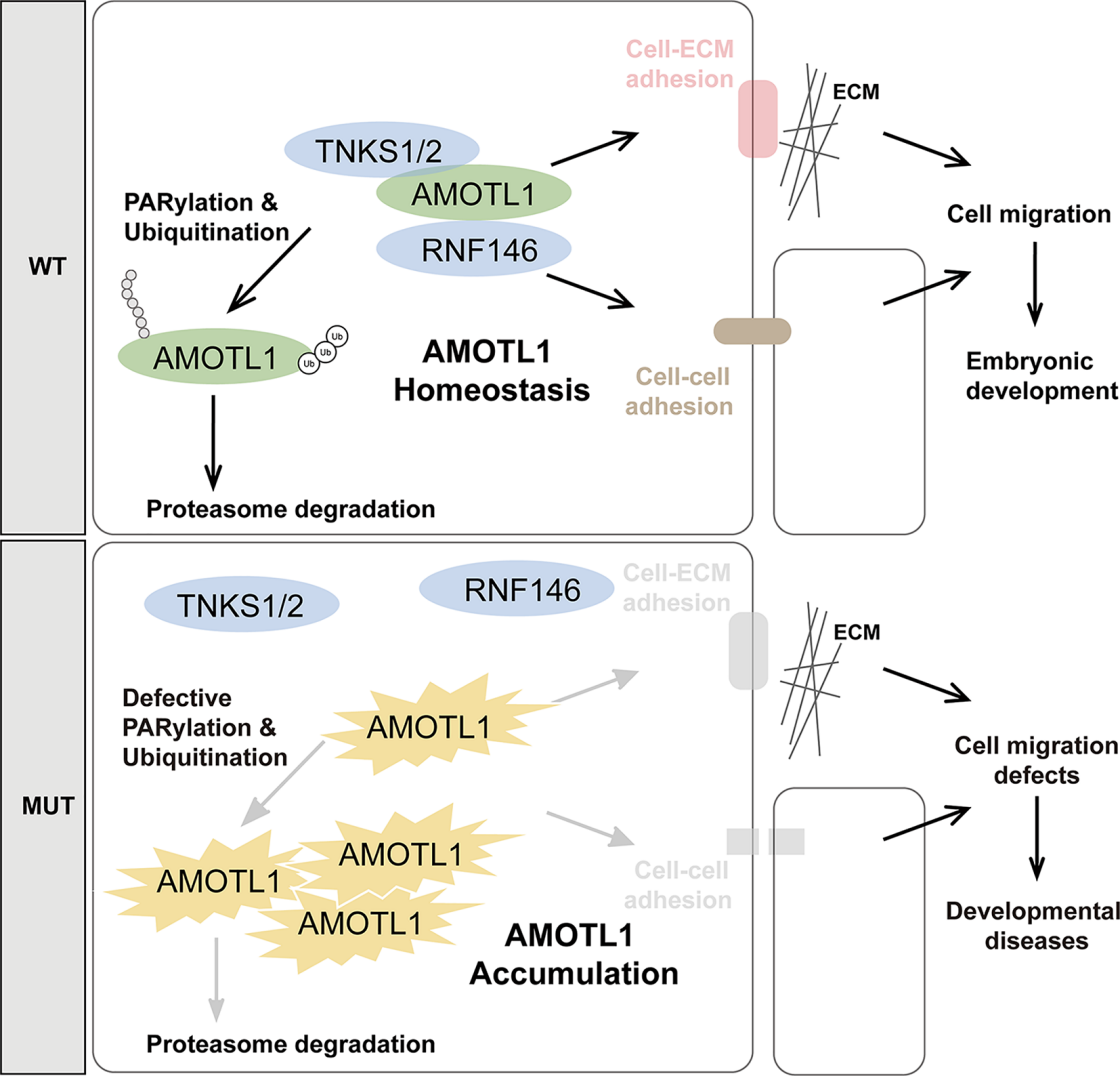
Schematic representation of the pathogenic mechanism for AMOTL1 mutants R157C and P160L mutations in AMOTL1 prevent its interactions with TNKS1/2 and RNF146, thereby inhibiting PARylation, ubiquitination, and subsequent proteasomal degradation. The resulting accumulation of AMOTL1 protein further disrupts cell–cell and cell–ECM adhesions, inhibits cell migrations, and contributes to developmental defects.

It is striking that both patient-derived *AMOTL1* mutations, R157C and P160L, are positioned within the highly conserved TBM across all Motin family proteins, and that these two amino acids are key residues in defining TBM [19,32]. Our results demonstrate that R157C and P160L AMOTL1 fail to interact with TNKS1/2 and RNF146, two enzymes critical for the PARylation and ubiquitination of AMOTL1. Loss of these modifications prevents the subsequent proteasomal degradation of AMOTL1, leading to its accumulation in cells. At the cellular level, AMOTL1 mutants, which cannot undergo PARylation, show reduced localization at the cell membrane and form abnormal aggregates within the cytoplasm, which may compromise cell–cell adhesions. Concurrently, their resistance to DDI2-dependent cleavage disrupts FAs, thereby weakening both cell–cell and cell–ECM adhesions. Cell–cell and cell–ECM adhesions are prerequisites for coherent cell migration. As a result, we observed that R157C AMOTL1 expression significantly inhibited both the speed and persistence of migrating cells. Given that the P160 is also essential for the interaction between Motins and TNKS1/2 and that the P160L mutation similarly stabilizes AMOTL1, P160L likely disrupts AMOTL1-mediated regulation of cell adhesion and migration through a comparable mechanism. Previous studies have shown that AMOTL1 deficiency loosens cell–cell junctions and promotes angiogenic sprouting [15], while overexpression of Motins or TNKS1/2 inhibition also disrupts cell–cell junctions [17]. Consistent with these findings, our results show that AMOTL1 overexpression leads to disorganized F-actin, impaired cell–cell adhesion, and defective cell–ECM adhesion (Figure 6). Collectively, these observations suggest that proper cellular levels of AMOTL1, and likely other Motins, are critical for maintaining cell junctions, the actin cytoskeleton, and FAs.

Active neural crest cell migration plays an important role in the development of craniofacial cartilages, heart, and skeletal muscle [41]. CNCCs migrate to form the embryonic orofacial cartilage, including the lower jaw that prefigures the adult craniofacial skeleton [38]. Consistently, we observed underdeveloped craniofacial cartilages and a shortened lower jaw in *AMOTL1-R157C*-injected zebrafish. However, neither the injected zebrafish nor the mouse model carrying the R157C variant exhibited phenotypes comparable to cleft lip or palate reported in affected human patients [27]. One possibility is that AMOTL1 variants exert more severe pathogenic effects in animal models, which may lead to embryonic lethality, as supported by the increased embryonic death rates of heterozygous and homozygous *Amotl1*-mutant mice [27]. Alternatively, the absence of overt clefting defects may reflect functional compensation from other developmental pathways, such as Shh or BMP signaling [42,43].

Notably, the severity of the cardiac and trunk phenotypes differs between zebrafish expressing WT AMOTL1 and the R157C mutant. This may reflect the higher protein stability of R157C AMOTL1, although we cannot exclude the possibility that the mutant is involved in additional pathogenic mechanisms, which warrants further investigation. In addition, microinjection of RNA into developing zebrafish embryos has inherent limitations, as RNA is relatively unstable and AMOTL1 expression is transient. Future studies using stable transgenic zebrafish lines will be instrumental in understanding the pathogenic functions of AMOTL1 during organogenesis in a more physiological context.

## Materials and methods

### Antibodies, plasmids, and other materials

Antibodies used in the present study are listed in Supplementary Table S1. Human WT and R157C mutated *AMOTL1*, *TNKS1*, and *RNF146* were cloned from a human complementary DNA (cDNA) library and subcloned into pLVX vector (Clontech) with indicated tags by homologous recombination. Primers used to generate *AMOTL1* point mutations are listed in Supplementary Table S2. All plasmids were confirmed by Sanger sequencing. CHX was purchased from Sigma–Aldrich (#C7698). BTZ was ordered from Selleck Chemicals (#S1013). Culture-Insert 2 Well (#80209) and μ-dish 35 mm (#81176) were purchased from Ibidi. Smart-Lifesciences anti-Flag beads (#SA042001) and Repligen Protein A agarose (#IPA300) were used for immunoprecipitation.

### Cell culture, transfection, and lentivirus transduction

Human embryonic kidney cells (HEK293A and HEK293T) and MDCK were cultured in DMEM (Corning) containing 10% FBS (Gibco) and 1x penicillin/streptomycin (P/S, Meilunbio) at 37°C under 5% CO2. Transfections were performed using PolyJet DNA In Vitro Transfection Reagent (Signagen Laboratories) following the manufacturer’s instructions. HEK293 and MDCK cells stably expressing Vector, WT, or mutated AMOTL1 were obtained through lentivirus infection. Lentivirus was produced by co‐transfecting HEK293T cells with viral vectors and packaging plasmids (psPAX.2 and pMD2.G). After 48 h of transfection, the lentivirus supernatant was filtered through a 0.45‐μm filter, followed by infecting cells in the presence of 5 μg/ml polybrene for 16 h. Cells were then cultured in fresh complete medium for 24 h and then selected with 1 μg/ml puromycin (Invivogen).

### RNA extraction and real-time quantitative PCR

Total mRNA was extracted and purified using TAKARA MiniBEST Universal RNA Extraction Kit following the manufacturer’s instructions. The cDNA was synthesized using TAKARA PrimeScript™ RT Master Mix. RT-PCR was carried out using ABI 7500 FAST instrument and TAKARA SYBR Premix Ex Taq (Tli RNaseH Plus) kit. The expression of GAPDH mRNA was used as reference. Primers were synthesized by Tsingke Biotech, China. Experiments were repeated at least three times, and primers (sequences in the 5′–3′ direction) used in the present study are as follows:

human *AMOTL1*: TGGAGGGCAAAGTTGCGCCG (F), GCTGGTCCTGCACTCCCTGT (R);
human *GAPDH*: ATGGGGAAGGTGAAGGTCG (F), GACCACCTGGTCCTCAGTGT (R);
canine *GAPDH*: AACATCATCCCTGCTTCCAC (F), GACCACCTGGTCCTCAGTGT (R).

### Immunoblotting

Cells were lysed in 1× SDS loading buffer containing 50 mM Tris (pH 6.8), 2% SDS, 0.025% bromophenol blue, 10% glycerol, and 5% β-mercaptoethanol. The concentration of total proteins was determined by the BCA method. Proteins were separated by SDS–polyacrylamide gel electrophoresis and then transferred onto polyvinylidene fluoride membranes. The membranes were blocked with 5% milk in TBST at room temperature for 1 h and immunoblotted with primary antibodies at 4°C overnight. Afterward, the membranes were incubated with HRP-conjugated secondary antibodies for 1 h and followed by extensive washing and treatment with enhanced chemiluminescence solution. Signals were detected and recorded using a 5200S Imager (Tanon). ImageJ software was used for protein quantification.

### Co-immunoprecipitation

HEK293T cells were seeded in six-well plates and transiently transfected with plasmids using PolyJet (SignaGen). Cells were washed with ice-cold PBS and then lysed on ice using mild lysis buffer containing 20 mM Tris, 2 mM EDTA, 100 mM NaCl, 1% NP‐40, 50 mM NaF, and 1 mM Na_3_VO_4_, with PMSF (1 mM) and protease inhibitor cocktails (Selleck) added freshly before use. Lysates were clarified by centrifugation (10,000×***g***; 4°C; 10 min), and supernatants were incubated at room temperature for 1 h with anti-Flag beads or anti-Ha primary antibodies (Cossen Biotech, #2016BD0101006) and followed by incubation with Protein A agarose for 1 h. Beads were washed with lysis buffer four times, and bound proteins were eluted in 1× SDS loading buffer and subjected to immunoblotting.

### Immunofluorescence and confocal microscopy

MDCK cells seeded on coverslips or Culture-Inserts were fixed with 4% paraformaldehyde for 15 min at room temperature, and permeabilized in 0.1% Triton X-100 for 10 min. Fixed cells were blocked with 3% BSA and 3% goat serum in PBS for 1 h and incubated with primary antibody overnight at 4°C. After washing with PBS three times, cells were incubated in Alexa Fluor 555- or 647-conjugated secondary antibodies for 1 h at room temperature. Cells were then washed with TBS three times and mounted. Images were captured using a ZEISS LSM 900 with Airyscan 2 confocal microscope with a 63× Plan-Apochromat lens. Zeiss ZEN Lite software and ImageJ (National Institutes of Health) software were used to acquire and process images.

### Cell migration assay

MDCK cells were seeded at 5 × 10^4^ cells/well into Culture-Insert mounted in a dish. When cells reached a dense monolayer, Culture-Inserts were removed and the medium was replenished. Cell migration into the open space (wound) created was monitored for 24 h using an Olympus scanR high-content screening station. Each evaluated sample was plated in triplicate. Videos were processed with ImageJ software. Wound closure was measured at different time points and expressed as percentages of the initial wound area.

### Zebrafish husbandry and microinjection

WT (AB) zebrafish were maintained at 28.5°C under a 14 h light/10 h dark cycle. Embryos were raised at the same temperature in standard embryo medium. For anesthesia, zebrafish were treated with tricaine (MS-222, 100–200 mg/l, pH 7.0) until loss of response, and killed by prolonged exposure to a high concentration of MS-222 (>500 mg/l). All experiments were performed at Fudan University (Shanghai, China) in accordance with institutional and national guidelines, and were approved by the Institutional Animal Care and Use Committee of Fudan University.

### Zebrafish microinjection

Full-length human *AMOTL1* and *AMOTL1* R157C cDNAs were cloned into pCS2 vector. To confirm microinjection efficiency, the mCherry gene and E2A sequences were inserted upstream of *AMOTL1*/*AMOTL1* R157C. mRNA was synthesized by *in vitro* transcription using the mMESSAGE mMACHINE kit (Ambion) and purified using MEGAclear kit (Invitrogen) according to the manufacturer’s instructions. A total of 450 pg of each mRNA was injected into one-cell stage embryos as described [44].

### Zebrafish staining and imaging

Alcian blue or phalloidin staining was carried out as previously described [45,46]. Bright-field, fluorescence, and Alcian blue staining images were captured using a Leica M205FA microscope. The images of phalloidin staining were taken using Olympus FV3000 microscope.

### Calculation of heartbeat rate and phenotype

Embryos were embedded in 1% low melting agarose for calculation of heartbeat. Heartbeat frequency was calculated for 3 min under the microscope. Larvae were divided into three groups based on the appearance of their trunks: severe phenotype, moderate phenotype, and normal phenotype. The percentage of each phenotype was calculated by dividing the number of larvae in each group by the total number of larvae.

### Mutation pathogenicity prediction

In the present study, the SIFT (http://sift.jcvi.org), PROVEAN (http://provean.jcvi.org/), Polyphen2 (http://genetics.bwh.harvard.edu), and MutationTaster (http://www.mutationtaster.org/) methods were used to predict the effects of amino acid substitution on the protein function [47–50].

### Statistical analysis

All experiments were performed with at least three independent replicates, each with technical replicates to ensure reproducibility. Data are presented as mean ± SEM unless otherwise specified. Comparisons between groups were analyzed for statistical significance with the two-tailed Student’s *t*-test. The Chi-square test for trend was used to evaluate difference in the distribution of embryonic phenotypes across experimental groups. Statistical analyses were carried out using Prism Version 8.0 (GraphPad Software Inc). Statistical significance was defined as follows: not significant (ns), *P* <0.05 (*), *P* <0.01 (**), *P* <0.001 (***), and *P* <0.0001 (****).

## Supplementary Material

Supplementary Figures S1-S3 and Tables S1-S2

Supplementary Video S1

## Data Availability

No datasets were used and analyzed in the current study.

## Abbreviations

AMOT: Angiomotin
AMOTL1: Angiomotin-like 1
AMOTL2: Angiomotin-like 2
BTZ: bortezomib
BCA: bicinchoninic acid
CB: ceratobranchial
CCM: collective cell migration
cDNA: complementary DNA
CH: ceratohyal
CHX: cycloheximide
CNCC: cranial neural crest cell
DAPI: 4′,6-diamidino-2-phenylindole
ECM: extracellular matrix
F-actin: filamentous actin
FA: focal adhesion
FAK: focal adhesion kinase
FBS: fetal bovine serum
GAPDH: glyceraldehyde-3-phosphate dehydrogenase
GFP: green fluorescent protein
HEK: human embryonic kidney
HRP: horseradish peroxidase
HSP90: heat shock protein 90
LATS1/2: large tumor suppressor kinase 1/2
LPA: lysophosphatidic acid
MDCK: Madin–Darby canine kidney
ME: Meckel’s cartilage
MOTINS: Motin family (AMOT, AMOTL1, AMOTL2)
NF2: neurofibromin 2
OE: overexpression
PARylation: poly ADP-ribosylation
PMSF: phenylmethylsulfonyl fluoride
PQ: palatoquadrate
RNF146: Ring finger protein 146
TBM: Tankyrase-binding motif
TBST: Tris-buffered saline with Tween 20
TNKS: Tankyrase
UPS: ubiquitin–proteasome system
WT: wild-type
ZO-1: zonula occludens-1
