# Supplementary material for: Patient-derived AMOTL1 mutations lead to defective cell migration and tissue development
Source: Biosci Rep. 2026 May 11;46(5):BSR20250149. doi: 10.1042/BSR20250149 (PMC13161331; doi:10.1042/BSR20250149)
Supplement: Supplementary Figures S1-S3 and Tables S1-S2 [file BSR-2025-0149_supp.pdf]

Supplementary Figure Legends

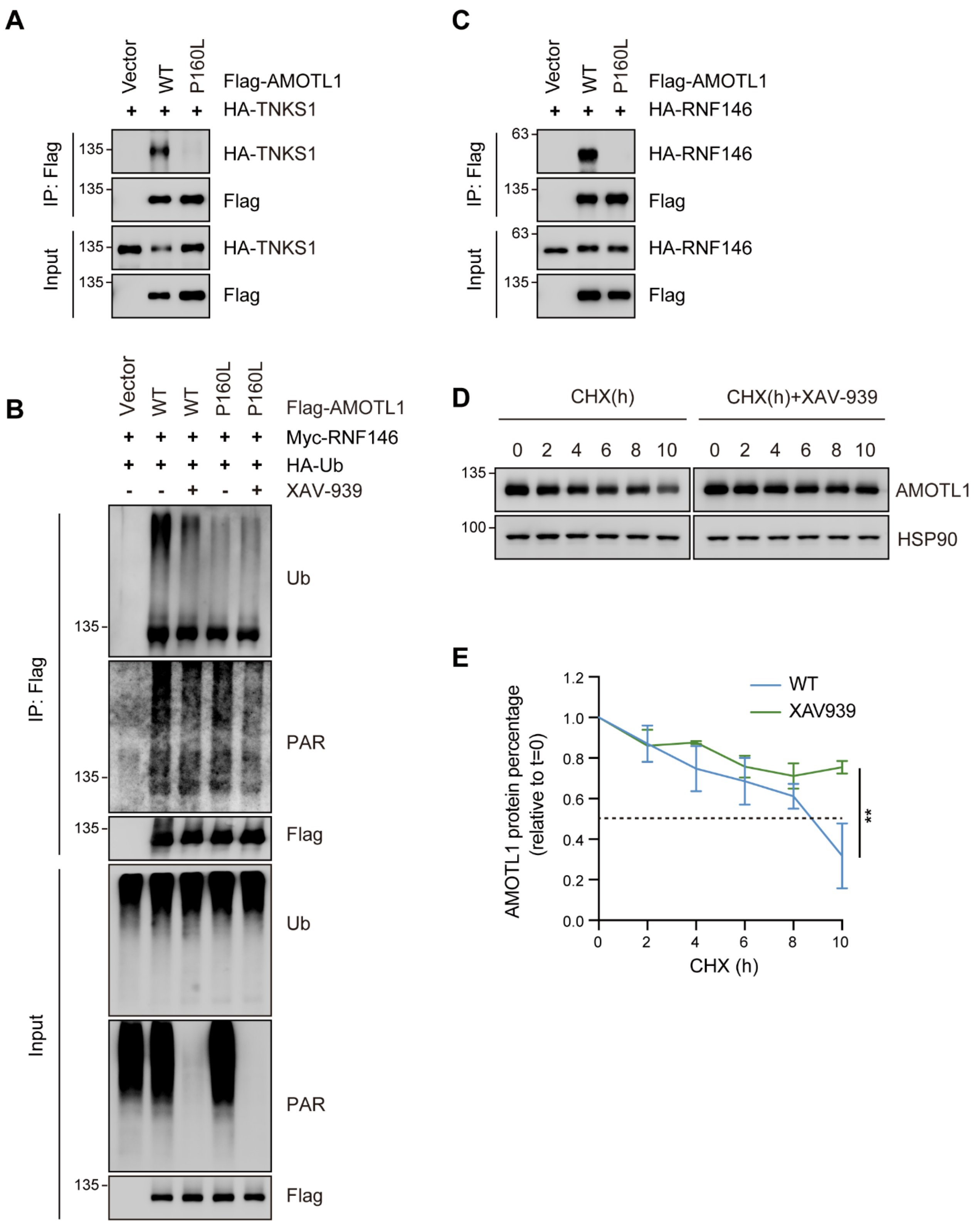

Figure S1. P160L mutation impairs TNKS–RNF146–dependent modification and destabilization of AMOTL1.

(A, B) Co-immunoprecipitation showing that the P160L mutation of AMOTL1 disrupts its interaction with TNKS1 (A) and RNF146 (B).

(C) The P160L mutation reduces PARylation and ubiquitination of AMOTL1. Cells expressing Flag-AMOTL1 (WT or P160L) were treated with the tankyrase inhibitor XAV-939 as indicated, followed by anti-Flag immunoprecipitation and immunoblotting.

(D, E) Inhibition of tankyrase stabilizes AMOTL1 protein. Cycloheximide (CHX) chase assay showing AMOTL1 protein levels over time in the absence or presence of XAV-939 (D), with quantification shown in (E). Data are presented as mean ± SEM; $^{**}P < 0.01$.

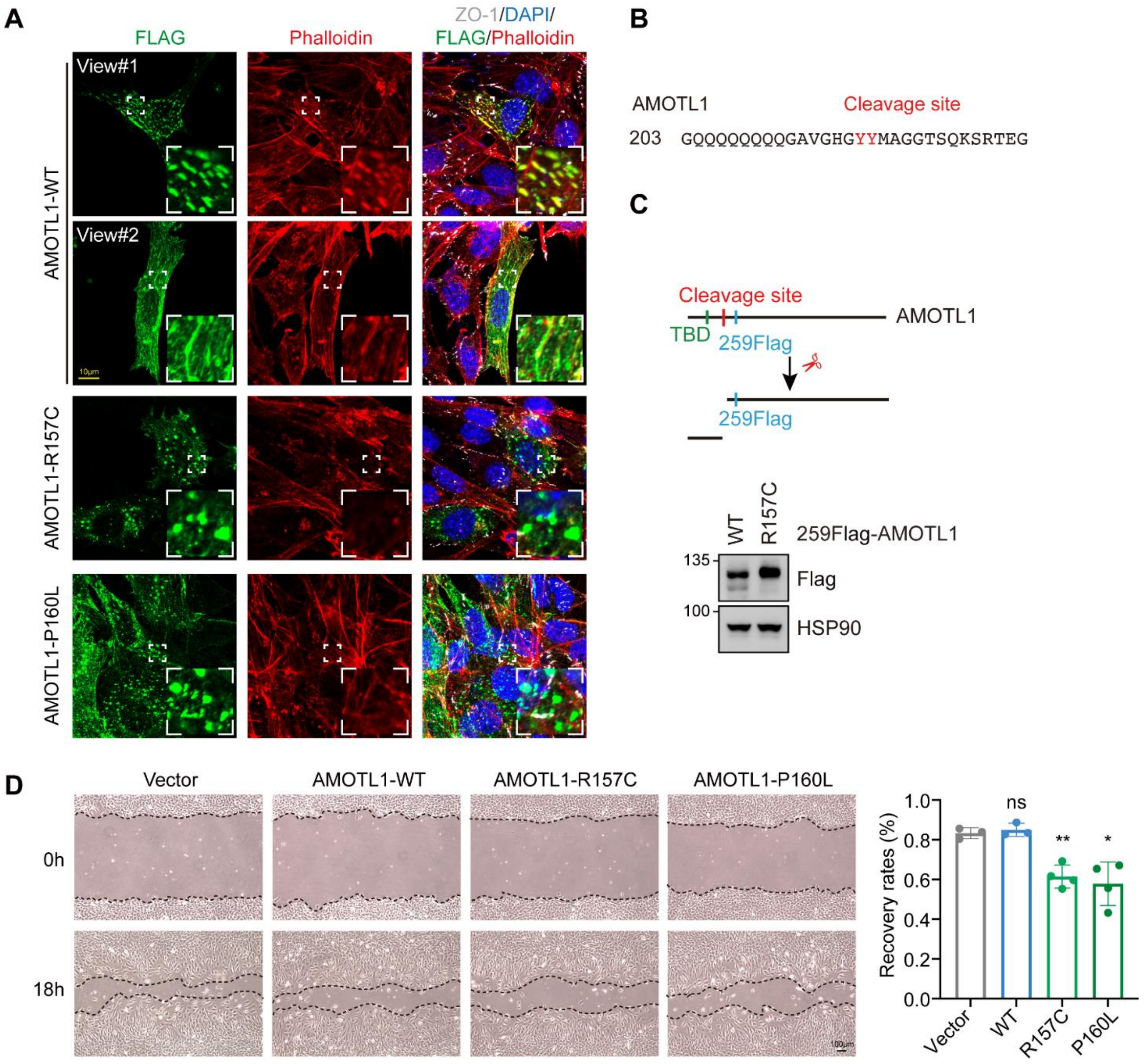

Figure S2. AMOTL1 patient-derived mutants fail to associate with actin stress fibers and resist proteolytic cleavage.

(A) Representative confocal images showing the subcellular localization of Flag-tagged AMOTL1 WT, R157C, and P160L. Cells were stained for Flag (green), F-actin (phalloidin, red), ZO-1 (white), and nuclei (DAPI, blue). Insets show magnified views of the boxed regions. Scale bar, 10 µm.

(B) Schematic representation of the putative cleavage site within AMOTL1.

(C) Schematic illustrating the strategy for detecting AMOTL1 cleavage using an internal Flag tag (259Flag) positioned downstream of the cleavage site, and immunoblot analysis showing that the R157C mutation impairs AMOTL1 cleavage. HSP90 was used as a loading control.

(D) Wound healing assay showing migration of MDCK cells expressing Vector control, AMOTL1-WT, AMOTL1-R157C, or AMOTL1-P160L. Representative images were taken at 0 h and 18 h after removal of the culture insert. Dashed lines indicate the wound edges. Right: Quantification of wound recovery rates. Data are presented as mean ± SEM. ns, not significant; *$P < 0.05$, **$P < 0.01$ (two-tailed Student's t-test).

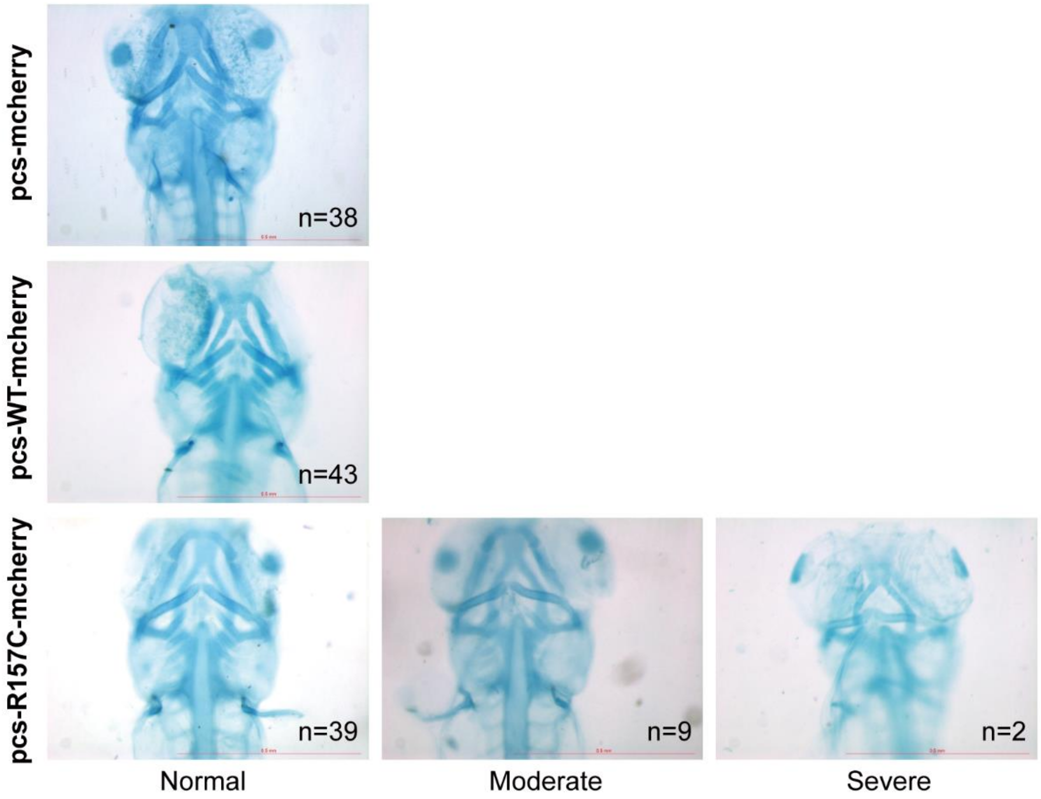

Figure S3. Classification of craniofacial cartilage phenotypes in zebrafish expressing AMOTL1. Representative Alcian blue staining of craniofacial cartilage in zebrafish embryos at 3 days post fertilization (dpf). Embryos expressing AMOTL1 R157C-mCherry (pcs-R157C-mCherry) displayed craniofacial abnormalities with variable severity. The phenotypes were categorized into three groups based on cartilage morphology. Normal: embryos showing overall intact craniofacial cartilage structures comparable to control embryos. Moderate: embryos exhibiting partial defects in craniofacial cartilage organization, including altered ceratohyal (CH) morphology or reduced development of anterior or branchial arch structures. Severe: embryos displaying pronounced craniofacial cartilage defects, including strong disruption or loss of multiple cartilage elements.

Supplementary Table 1. Information about antibodies used in this article. CST: Cell Signaling Technology; BD: BD Pharmingen; Santa Cruz: Santa Cruz Biotechnology; SIGMA: Sigma-Aldrich Scientific; Thermo: Thermo Fisher; MBL: MBL Life science.

**Antibody**	**Company**	**Catalog**	**Dilution**	
			**IB**	**IF**
**Flag-HRP**	Sigma	A8592	1:10,000	
**Ha-HRP**	CST	14031	1:5,000	
**Myc**	MBL	M192-3	1:10,000	
**PAR**	Trevigen	4336-BPC-100	1:1,000	
**NF2**	CST	12888S	1:2,000	
**HSP90**	BD	610418	1:10,000	
**FAK**	CST	71433T		1:100
**Phalloidin**	Invitrogen	A22287		1:100
**ZO-1**	Thermo	1A12	1:1,000	1:100

Supplementary Table 2. Primers used to generate AMOTL1 point mutations.

Primer	Sequence
hAMOTL1-R157C-frag1-F	TTCTGTTCCAGGGGCCCACCGGTATGTGGAGGGCAAAGTT
hAMOTL1-R157C-frag1-R	TGACCCTGCGGTTCTTGGCATGCTGACTGGTAGACCATTT
hAMOTL1-R157C-frag2-F	AATGGTCTACCAGTCAGCATGCCAAGAACCGCAGGGTCAA
hAMOTL1-R157C-frag2-R	CTCCAGCGAATTGGCGAATTCTTAGATGAGGACTTCCATCATC
hAMOTL1-P160L-frag1-F	TTCTGTTCCAGGGGCCCACCGGTATGTGGAGGGCAAAGTT
hAMOTL1-P160L-frag1-R	CTGGTGTTCTTGACCCTGCAGTTCTTGGCGTGCTGACTGG
hAMOTL1-P160L-frag2-F	GCACGCCAAGAACTGCAGGGTCAAGAACACCAGGTGGACA
hAMOTL1-P160L-frag2-R	CTCCAGCGAATTGGCGAATTCTTAGATGAGGACTTCCATCATC

Supplementary Video. Live-cell imaging of migrating control MDCK cells or cells expressing WT or R157C AMOTL1 over 24 h.
